# Predictive Biomarkers of Gastroesophageal Reflux Disease and Barrett’s Esophagus in World Trade Center Exposed Firefighters: a 15 Year Longitudinal Study

**DOI:** 10.1038/s41598-018-21334-9

**Published:** 2018-02-15

**Authors:** Syed H. Haider, Sophia Kwon, Rachel Lam, Audrey K. Lee, Erin J. Caraher, George Crowley, Liqun Zhang, Theresa M. Schwartz, Rachel Zeig-Owens, Mengling Liu, David J. Prezant, Anna Nolan

**Affiliations:** 1 Department of Medicine, Division of Pulmonary, Critical Care and Sleep Medicine, New York University School of Medicine, New York, NY USA; 2 Department of Environmental Medicine, New York University School of Medicine, New York, NY USA; 3 Department of Population Health, Division of Biostatistics, New York University School of Medicine, New York, NY USA; 4 Bureau of Health Services, Fire Department of New York, Brooklyn, NY USA; 5 Department of Respiratory Medicine, PLA, Army General Hospital, Beijing, China; 6 Department of Medicine, Pulmonary Medicine Division, Montefiore Medical Center and Albert Einstein College of Medicine, Bronx, NY USA

## Abstract

Gastroesophageal reflux disease (GERD) and Barrett’s Esophagus (BE), which are prevalent in the World Trade Center (WTC) exposed and general populations, negatively impact quality of life and cost of healthcare. GERD, a risk factor of BE, is linked to obstructive airways disease (OAD). We aim to identify serum biomarkers of GERD/BE, and assess the respiratory and clinical phenotype of a longitudinal cohort of never-smoking, male, WTC-exposed rescue workers presenting with pulmonary symptoms. Biomarkers collected soon after WTC-exposure were evaluated in optimized predictive models of GERD/BE. In the WTC-exposed cohort, the prevalence of BE is at least 6 times higher than in the general population. GERD/BE cases had similar lung function, D_**LCO**_, bronchodilator response and long-acting β-agonist use compared to controls. In confounder-adjusted regression models, TNF-α ≥ 6 pg/mL predicted both GERD and BE. GERD was also predicted by C-peptide ≥ 360 pg/mL, while BE was predicted by fractalkine ≥ 250 pg/mL and IP-10 ≥ 290 pg/mL. Finally, participants with GERD had significantly increased use of short-acting β-agonist compared to controls. Overall, biomarkers sampled prior to GERD/BE presentation showed strong predictive abilities of disease development. This study frames future investigations to further our understanding of aerodigestive pathology due to particulate matter exposure.

## Introduction

The destruction of the world trade center (WTC) complex lead to the exposure of thousands of first responders and inhabitants of New York City to WTC-particulate matter (WTC-PM)^1–6^. WTC-PM exposure in our Fire Department of New York (FDNY) cohort is associated with the development of obstructive airways disease (OAD), gastroesophageal reflux disease (GERD) and Barrett’s Esophagus (BE)^7–9^. By 2005, approximately 44% of WTC rescue and recovery workers had developed GERD symptoms, which is 8.2 times its pre-9/11 prevalence^10^. GERD symptoms are reported by 20% of the US population^11,12^. The incidence of GERD in the US is approximately 5 per 1000 person-years^13,14^, and GERD-related disease accounts for as much as 5% of all outpatient visits^15,16^. In short, there is diminished health-related quality of life and productivity associated with GERD^15–17^. GERD is an independent risk factor in the development of the metaplastic changes of BE^18^, which can lead to adenocarcinoma^19^. GERD is also associated with occupational or environmental exposure related OAD^8,20,21^.

Overall, WTC-exposed firefighters with OAD had a three times higher risk of developing GERD^22^. In WTC-exposed adults, persistent GERD occurred more often in participants with asthma^9^. Although many investigators have suggested interdependence between airway and digestive diseases, the causative factors and specific mechanisms remain unclear^23^. We have successfully identified metabolic, vascular and inflammatory biomarkers of WTC-Lung Injury (WTC-LI)^20,21,24–27^. Identification of biomarkers of GERD/BE in a population with respiratory disease may facilitate identification of biologically relevant immune pathways.

We hypothesize that serum biomarkers obtained within 200 days after exposure to WTC particulates will be different in FDNY rescue and recovery workers who proceed to develop GERD/BE. Therefore, the objectives of this study are to (i) determine predictive biomarkers of GERD/BE and to (ii) describe the respiratory and clinical characteristics of participants with GERD/BE in this population of WTC-exposed first responders^28–30^.

## Results

### Demographics and Phenomics

**Figure 1:**
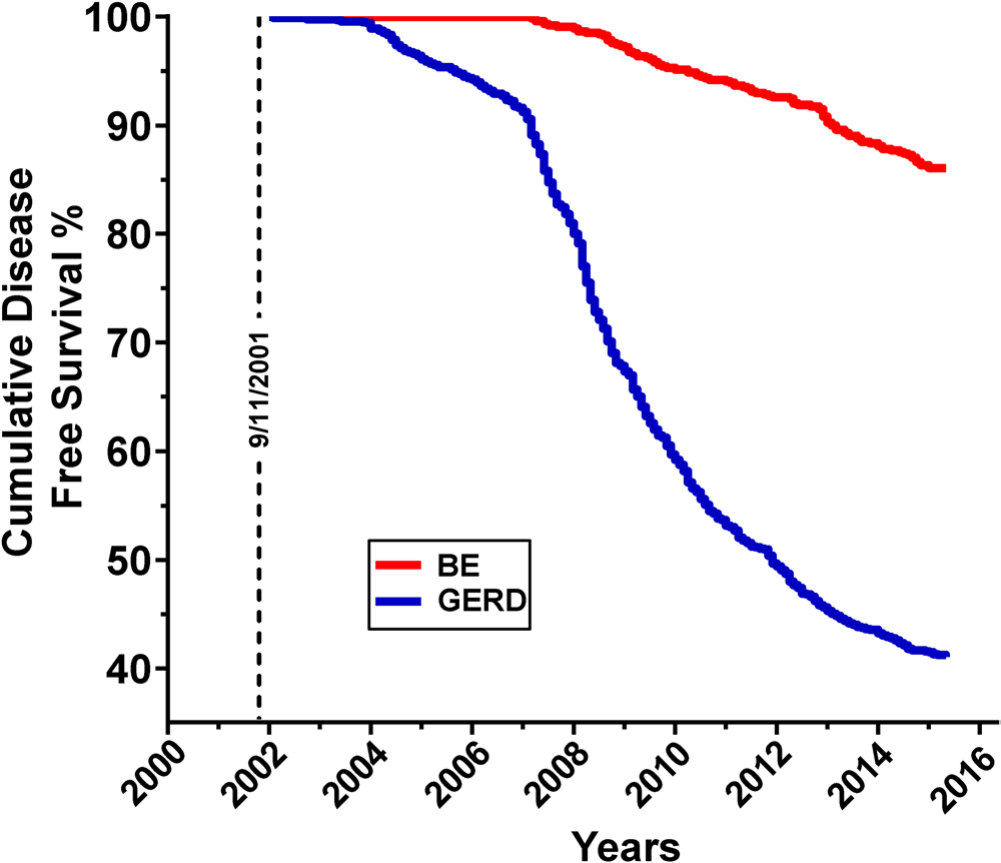
GERD/BE Disease Free Survival. Participants were followed for 15 years. *GERD = Gastroesophageal Reflux Disease, BE = Barrett’s Esophagus.

Participants attended annual physical exams until 01/18/2015. Prevalence of GERD and BE in the source cohort is 58.8% and 6.8% respectively, while the incidence of GERD and BE is 60.19 and 5.06 cases per 1000 person–years, respectively. All BE cases in this cohort had a prior diagnosis of GERD. Disease free survival curves of GERD and BE were significantly different (p < 0.0001) (Fig. 1). There was no significant difference in age and exposure intensity between cases of GERD and BE, and their controls. Body mass index (BMI) was significantly different between cases (GERD/BE) and controls at the time of enrollment in the Medical Monitoring and Treatment Program (MMTP), as well as subspecialty pulmonary evaluation (SPE), and was therefore considered in logistic regression analyses. The total duration of exposure at the site was not different between cases of GERD/BE and controls. Lung function as measured by forced expiratory volume in 1 second (FEV_1_), forced vital capacity (FVC) and FEV_1_/FVC at the time of symptomatic presentation to SPE was not different in those with GERD, BE, or when either was compared to controls, Table 1.

Of participants with prescription data in the biomarker available group, 68% (144/219) were prescribed short-acting β-agonist (SABA). Among the SABA using participants, 63% (91/144) developed GERD. Fifty-one percent of the remaining participants with no documented SABA use (38/75) developed GERD. SABA use was significantly increased in GERD cases compared to controls in the source cohort (p = 0.048). Of participants with prescription data in the biomarker available group, 17% (36/215) were prescribed long-acting β-agonist (LABA). Among the LABA using participants, 69% (25/36) developed GERD. Fifty-seven percent of the remaining participants with no documented LABA use (102/179) developed GERD. LABA use was not significantly different between GERD cases and controls in both cohorts. There was no significant association between SABA or LABA use and BE.

### GERD Biomarker Model Development

Initially, 15 biomarkers were assessed as continuous variables for their predictive ability using Mann-Whitney *U* test. C-peptide (p = 0.01), MMP-9 (p = 0.01), diastolic blood pressure (BP) (p = 0.01) and BMI (p = 0.02) were statistically significant between GERD and controls. At a false discovery rate (FDR) of 0.15, MMP-9, C-peptide, Systolic and Diastolic BP, and BMI were identified as differentially expressed between GERD and control groups (Supplementary Table S1). *Crude Model*: Tumor necrosis factor-alpha (TNF-α) ≥ 6 pg/mL and C-peptide ≥ 360 pg/mL significantly predicted GERD in the univariate model. The *Full Model* was adjusted for potential confounders including age at 9/11, BMI at the time of MMTP/serum collection and WTC-PM exposure intensity. TNF-α ≥ 6 pg/mL and C-peptide ≥ 360 pg/mL both remained significant predictors of GERD in the confounder-adjusted final multivariate model with odds ratios [OR(95%CI)] of 2.06(1.15–3.70) and 2.08(1.20–3.61), respectively (Table 1).

**Table 1:** Clinical Measures, Biomarker Prevalence and Model Definition.

Clinical Measure		Source Cohort			Biomarker Cohort			Odds Ratio			
		GERD N = 915	Controls N = 637	BE N = 106	GERD N = 153	Controls N = 112	BE N = 20	Crude		Full Model^a^	
								GERD	BE	GERD	BE
**Caucasian**		**846** (92.5)	**581** (91.2)	**97** (91.5)	**147** (96.1)	**109** (97.3)	**18** (90)	**0.67** (0.17–2.76)	**0.25** (0.04–1.59)		
**Duration** **(months)**		**3** (1–6)	**2** (1–5)	**3** (1–7)	**2** (1–6)	**3** (1–5)	**3** (2–7)	**0.99** (0.91–1.09)	**1.14** (0.98–1.34)		
**PFT at SPE**	**FEV**_1_	**90** (80–99)	**89** (80–98)	**91** (81–100)	**91** (81.0–102.5)	**93.5** (83.3–104.0)	**92.5** (85–107)	**1.00** (0.98–1.01)	**1.00** (0.97–1.03)		
	**FVC**	**86** (77–94)	**85** (76–94)	**87** (79–98)	**88** (79–97)	**88** (80–97)	**90** (81.3–98.5)	**1.00** (0.99–1.02)	**0.65** (0.27–1.52)		
	**FEV**_1_ **/FVC**	**83.8** (79.3–87.0)	**84.0** (79.5–87.4)	**83.7** (79.1–86.8)	**83.7** (78.8–87.0)	**84.6** (80.6–87.4)	**85** (82.5–88.7)	**0.97** (0.93–1.01)	**1.01** (0.93–1.10)		
**BMI**	**MMTP Entry**	**28.4** (26.4–30.7)	**28** (26.0–30.3)	**28.5** (26.6–30.9)	**28.1** (26.0–30.7)	**27.8** (25.8–30.1)	**27.6** (25.9–29.8)	**1.05** (0.97–1.12)	**0.98** (0.86–1.12)	**1.02** (0.95–1.10)	**0.93** (0.81–1.08)
	**SPE**	**30.1** (27.4–33.3)	**29.4** (27.0–32.4)	**29.7** (27.5–33.5)	**29.4** (26.9–31.7)	**28.5** (26.5–31.1)	**28.9** (26.8–30.8)	**1.05** (0.99–1.11)	**0.99** (0.88–1.11)		
**Age on 9/11**		**41** (37–46)	**42** (36–46)	**43** (38–46)	**41** (37–46)	**40** (36.3–44.8)	**41** (37.3–46.0)	**1.01** (0.97–1.04)	**1.02** (0.95–1.09)	**0.99** (0.95–1.03)	**1.01** (0.94–1.09)
**Exposure**	**Low**	**681** (71.1)	**491** (73.2)	**78** (70.9)	**125** (81.7)	**86** (76.8)	**14** (70.0)	**Reference**			
	**High**	**234** (24.4)	**146** (21.8)	**28** (25.5)	**28** (18.3)	**26** (23.2)	**6** (30)	**1.35** (0.74–2.46)	**0.71** (0.25–2.02)	**1.31** (0.70–2.43)	**0.55** (0.16–1.85)
**Biomarker**	**C-peptide** ^b^				**791.2** (372.3–1792.3)	**550.7** (250.5–1305.8)	**755.6** (436.9–1176.5)	**2.12** (1.24–3.62)		**2.08** (1.20–3.61)	
	**TNF-α** ^b,c^				**4.7** (2.9–6.9)	**4.3** (2.9–5.7)	**5.7** (3.5–6.8)	**2.09** (1.19–3.68)	**2.90** (1.09–7.72)	**2.06** (1.15–3.70)	**3.84** (1.23–12.03)
	**Fractalkine**				**63.7** (28.4–155.6)	**70.6** (26.4–142.6)	**101.4** (39.7–597.1)		**3.96** (1.46–10.76)		**3.42** (1.18–9.96)
	**IP-10** ^c^				**257.6** (200.2–350.4)	**236.6** (183.2–308.2)	**323.2** (236.7–662.5)		**3.95** (1.47–10.60)		**4.47** (1.45–13.84)

Median(IQR) or N(%) as indicated; FEV_1_ and FVC as % Predicted.

^a^Logistic Regression Model for biomarkers adjusted for age on 9/11, Exposure, and BMI at MMTP Entry.

Hosmer-Lemeshow goodness-of-fit, GERD = χ^2^, 5.958; df = 8; *P* = 0.65 & BE = χ^2^, 8.11; df = 8; *P* = 0.42. ^b^p < 0.05 Mann-Whitney *U* test GERD vs Controls; ^c^p < 0.05 Mann-Whitney *U* test BE vs Controls.

Biomarker Cutpoints: C-peptide ≥ 360 pg/mL, TNFα ≥ 6 pg/mL, Fractalkine ≥ 250 pg/mL, IP-10 ≥ 290 pg/mL.

TNF-α**-** Tumor Necrosis Factor-Alpha; IP-10**-** Interferon gamma-induced protein-10.

### BE Biomarker Model Development

Similarly, TNF-α (p = 0.02), IP-10 (p = 0.01), IL-6 (0.04) and Insulin (p = 0.01) were significant between BE and controls as continuous variables. TNF- α, IP-10, IL-6 and Insulin were also identified as significantly expressed biomarkers between BE and controls at an FDR of 0.15 (Supplementary Table S1). Predictive biomarkers of BE were also identified using crude and confounder-adjusted binary logistic regression models. *Crude Model*: TNF-α ≥ 6 pg/mL, fractalkine ≥ 250 pg/mL and interferon gamma induced protein-10 (IP-10) ≥ 290 pg/mL significantly predicted BE in the crude model. *Full Model*: The final multivariate model was adjusted for age at 9/11, BMI at the time of MMTP/Serum collection and WTC-PM exposure intensity. TNF-α ≥ 6 pg/mL, fractalkine ≥ 250 pg/mL and IP-10 ≥ 290 pg/mL continued to predict BE with ORs (95%CI) of 3.84(1.23–12.03), 3.42(1.18–9.96) and 4.47(1.45–13.84), respectively (Table 1). SABA and LABA use was not significant in any of our models, data not shown.

## Discussion

We identified serum biomarkers associated with the development of GERD and BE in a cohort of WTC-exposed FDNY firefighters with normal pre-9/11 lung function. These biologically plausible biomarkers of GERD and BE corroborate inflammation as a key contributor of GERD and pre-malignant BE. BE develops in response to chronic gastric reflux. Both GERD and BE are major risk factors for esophageal adenocarcinoma (EAC) as well^31,32^. In a case control study from Sweden, reflux symptoms were associated with EAC (OR 7.7); patients with long standing and severe symptoms were at greatest risk^33^. A meta-analysis concluded that at least weekly symptoms of GERD increased the odds of EAC fivefold, and further, daily symptoms increased the odds sevenfold^34^. Patients with BE have at least 30-fold higher risk of developing EAC than the general population. The absolute risk, however, is low, but higher in the presence of high-grade dysplasia^35^. The progression to EAC in BE depends on the type of dysplasia and it is important to note that the majority of BE patients will not develop carcinoma^36^. The identification of BE biomarkers is also significant for the clinically silent presentations (those without pre-BE development of GERD)^37^. The high mortality rate associated with esophageal cancer emphasizes the importance of the identification of biomarkers of potential esophageal cancer precursors.

Firefighters with elevated serum TNF-α and C-peptide within 6 months after exposure to WTC-PM had significantly greater odds of developing GERD, while those with elevated TNF-α, fractalkine and IP-10 had significantly greater odds of developing BE. BMI at the first visit to MMTP was significantly associated with GERD, but not BE. In the source cohort, there was no difference in lung function between cases and controls. Our final models were adjusted for BMI along with potential confounders, age, and PM exposure intensity. The duration of exposure was not significantly different in cases compared to controls. SABA use was significantly associated with GERD, but not BE. Interestingly, the prevalence of BE is almost six times higher in the source cohort than in the general population^37^. We thus support our hypothesis that cases of GERD and BE have different predictive serum biomarker profiles.

Interestingly, serum TNF-α was a biomarker of both GERD and BE. An increase in TNF-α expression occurs in esophageal epithelial cells during the metaplasia-dysplasia-carcinoma progression^38^. The observation that it predicts both GERD and BE in our cohort is promising. The mechanism of action and up-regulation of TNF-α in the evolution of BE and Barrett’s adenocarcinoma from esophageal inflammation has been explained in other studies^39,40^. Prior epidemiological studies have demonstrated a protective effect of aspirin and non-steroidal anti-inflammatory drugs against BE and esophageal adenocarcinoma^41^. Mechanism of this protective effect against BE may be through the inhibition of cyclooxygenase-2 (COX-2) and nuclear factor kappa-B (NF-κB) pathway which can be activated by pro-inflammatory cytokines including TNF-α^41,42^. Thus, the role of anti-TNF drugs in the management and prevention of BE needs to be determined.

We have observed elevated C-peptide predicting GERD in our cohort, opening a new discussion of involvement of insulin in the prediction of the GERD-BE disease cascade. Increased levels of insulin and insulin-like growth factor-1 (IGF-1) are associated with BE, and C-peptide is a known marker of insulin secretion^43^. Fractalkine (CX3CL1) is an inflammatory chemokine expressed in the peripheral blood and synovial fluid^44^. The CXCL10 gene in humans encodes IP-10. It is constitutively expressed at low levels in thymic, splenic, and lymph node stroma^45^. However, expression can be induced at high levels in monocytes, neutrophils, and endothelial cells, by stimulation with interferons (IFN-α, IFN-β, IFN-γ) or lipopolysaccharide (LPS), and in T cells by antigen activation^46–48^.

We also observed that SABA usage was significantly increased in participants with GERD, while there was no difference in BE cases. This was true despite the fact that cases of BE are a subset of those that developed GERD and that GERD and BE cases share TNF-α as a predictor. There may be several reasons for the difference in SABA use between cases of GERD and BE. The two groups may have had different symptoms clinically, leading to different treatment profiles for both their respiratory and gastrointestinal ailments. Prospective studies may allow us to further our understanding of SABA and LABA use in GERD and BE cases.

Increased prevalence of BE has been reported in veterans exposed to environmental toxins^49^. These findings resemble our rescue worker cohort that was similarly exposed to high concentrations of environmental dust. While prior studies have focused on histopathologic GERD biomarkers such as cell-to-cell adhesion molecules, dilated intercellular spaces, immunohistochemical markers and source intraluminal impedance, prior studies have not looked at PM exposed subjects^50^. The identification of biomarkers of GERD and BE in a PM-exposed cohort undergoing treatment for loss of FEV_1_ allows us to investigate the shared inflammatory pathways of aerodigestive disease. This investigation has identified that aerodigestive disease may be a result of shared inflammatory pathways of WTC-LI and GERD. Discovery of these biomarkers may allow for the early identification of individuals at risk for precancerous aerodigestive lesions such as BE.

There are several limitations to this study. Since the source cohort is composed of FDNY rescue workers with serum samples available in the first six months after 9/11/2001 and no pre-existing aerodigestive disease, the generalizability of these findings to other less well phenotyped cohorts is limited. The physician-documented diagnosis of GERD in the electronic medical record (EMR) was included as a case in our study. Therefore, not all of the GERD diagnoses in this study were based on endoscopic biopsies. We examined multiple biomarkers in our models, and concerns of multiple comparisons were addressed by the Benjamini-Hochberg method. Our study also lacks an unexposed control group. Therefore, we were only able to identify biomarkers of GERD and BE in the context of PM exposure. Replication of these findings in other longitudinally followed populations with and without PM exposure will be important to demonstrate the generalizability of these findings, especially the association of non-inflammatory biomarkers such as C-peptide. In addition, the temporality of OAD and GERD may be better explored in a prospective study. This would also allow for a better understanding of respiratory and GERD/BE treatment in relation to symptomatology. Our current study is also limited by the fact that we are only able to identify associations. Future work will include identifying biomarkers of GERD and BE in smokers and will include development of experiments that explore causality.

This is the first study that investigates predictive biomarkers of WTC-PM associated GERD and BE. We demonstrated that C-peptide predicts GERD, IP-10 and fractalkine predict BE, and TNF-α predicts both GERD and BE. Identification of these biomarkers may foster investigation into the pharmacological attenuation of biologically relevant pathways.

## Materials and Methods

### Study Design and Participants

**Figure 2:**
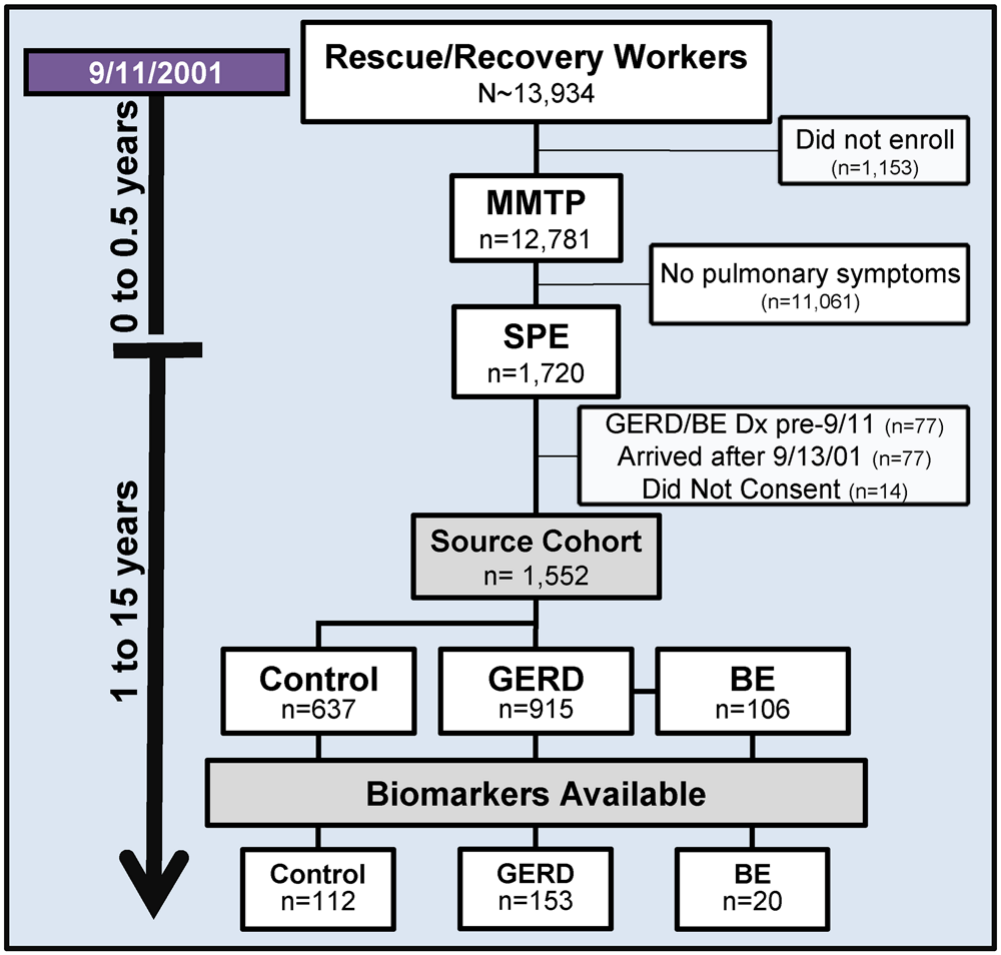
Study Design of FDNY Rescue Workers Exposed to World Trade Center Dust. Of the 13,934 exposed rescue and recovery workers, 92% enrolled in the MMTP. A subset (n = 1720) experienced pulmonary symptoms and an exclusion criterion was applied to form a source cohort (n = 1552). Blood biomarkers were analyzed in the source cohort. *GERD = Gastroesophageal Reflux Disease, BE = Barrett’s Esophagus

FDNY rescue and recovery workers were enrolled in the WTC-MMTP (N = 12,781) as previously described^21^. Participants in the source cohort (n = 1552) did not have a GERD diagnosis prior to 9/11/2001, had serum banked at MMTP (within 200 days of 9/11/2001), arrived at the WTC site before 9/13/01, and were members of the previously defined SPE cohort (Fig. 2)^24,27^. The EMR was used to identify cases of incident GERD and BE (until 2015). GERD cases (n = 915) were identified by review of post-9/11/2001 EMR and previously published literature^7,22,51–53^. BE cases (n = 106) were also identified by reported pathology on EMR. Cases were compared to randomly selected controls (n = 637) from the source cohort. Participants provided written informed consent and study was approved by the Institutional Review Boards of Montefiore Medical Center (#07-09-320) and New York University (#11-00439). All experiments were performed according to the relevant guidelines and regulations.

### Demographics

Age, gender, years of FDNY service and lung function measures were obtained from the FDNY WTC-EMR as previously described^21^. BMI was calculated from height and weight measured at MMTP and SPE. Degree of exposure was measured with respect to time of arrival at the WTC site on 9/11/2001 per the FDNY-WTC Exposure Intensity Index: i. Present on the morning of 9/11/2001 (high); ii. Arrived in the afternoon of 9/11/2001, up until 9/13/01 (low) as previously described (Table 1)^24–27,54,55^.

### Serum Sampling and Analysis

Biomarkers were assayed (n = 265) on a subset of the source cohort^21,24,26,54,56^. Serum of a representative subgroup of the source cohort with blood drawn at the time of enrollment in MMTP, consisting of cases of GERD (n = 153), BE (n = 20), and controls (n = 112), was analyzed. Samples were collected at MMTP between 10/29/2001 and 1/31/2002, serum was stored at −80 °C (Bio-Reference Laboratories, Inc. Elmwood Park, NJ), thawed once at 4 °C, and assayed using CVD-1 (HCVD1-67AK), Apo-lipoproteins (APO-62K), and Neurodegenerative (HNDG2-36K) panels per manufacturer’s instructions (Millipore, Billerica, MA) on a Luminex 200IS (Luminex Corporation). Data were analyzed with MasterPlex QT (Version 1.2; MiraiBio, Inc.) as previously described^26^.

### Statistical Analysis

SPSS 23 (IBM, Armonk, NY) was used for database management and statistics. Demographic information and analytes levels were compared by Mann-Whitney *U* test. Variables identified as potential confounders and those with a p-value < 0.2 between cases and controls were included in binary logistic regression analyses predicting case status (Table 1 and Supplementary Table S1). Benjamini-Hochberg procedure for multiple comparisons testing with FDR of 0.15 was used to determine statistical significance^57^. The maximum potential effectiveness of a biomarker was calculated by Youden Index^58^. Binary logistic regression analysis was used to calculate the odds ratios of GERD and BE biomarkers in crude and confounder-adjusted models. Goodness of fit of the model was evaluated using the Hosmer-Lemeshow test. Kaplan-Meier analysis was employed to assess the time of new onset of GERD and BE. The survival curves for both case groups were compared using the Log-rank test in Prism (v.7.01, GraphPad Software, La Jolla, California, USA), (Fig. 1). Pearson χ^2^-test was used to compare SABA and LABA usage between cases and controls. Significance was assessed by p < 0.05 for all statistical tests.

### Data availability

Dr. Nolan is the primary investigator and guarantor of the content of this manuscript, including the data and analysis. Sharing of human data is governed by the World Trade Center (WTC) Clinical Center of Excellence program maintained by the Fire Department of New York (FDNY). All investigators will need to enter into a data use agreement with the FDNY WTC Clinical Center of Excellence. Additional information about this database may be obtained through Dr. David Prezant. He can be reached by email at prezand@fdny.nyc.gov.

## Electronic supplementary material

Supplementary Table S1
