# Supplementary material for: Predictive Biomarkers of Gastroesophageal Reflux Disease and Barrett’s Esophagus in World Trade Center Exposed Firefighters: a 15 Year Longitudinal Study
Source: Sci Rep. 2018 Feb 15;8:3106. doi: 10.1038/s41598-018-21334-9 (PMC5814524; doi:10.1038/s41598-018-21334-9)
Supplement: Supplementary file 1 — Supplementary Table S1 [file 41598_2018_21334_MOESM1_ESM.doc]

**Supplemental Data**

**Predictive Biomarkers of Gastroesophageal Reflux Disease and Barrett’s Esophagus in World Trade Center Exposed Firefighters: a 15 Year Longitudinal Study**

Syed H. Haider, MD, Sophia Kwon, DO, MPH, Rachel Lam BA, Audrey K. Lee, BA, Erin J. Caraher, MD, MS, George Crowley, BA, Liqun Zhang, MD, PhD, Theresa M. Schwartz, MS, Rachel Zeig-Owens, MPH, PhD, Mengling Liu, PhD, David J. Prezant, MD, *Anna Nolan, MD, MS

| **Table S1. Biomarkers of GERD and BE** | | | | | | | | |
| --- | --- | --- | --- | --- | --- | --- | --- | --- |
| **Analytes** | | **p*** | **q**** | **GERD** | **Control** | **BE** | **p^†^** | **q**** |
| **TNF-α** | | 0.13 | 0.269 | **4.68**  (2.87-6.87) | **4.34**  (2.90-5.72) | **5.64**  (3.60-6.78) | 0.02 | 0.112 |
| **C-peptide** | | 0.01 | 0.056 | **789.65**  (372.37-1789.49) | **550.66**  (250.52-1305.82) | **798.37**  (443.20-1410.81) | 0.13 | 0.242 |
| **Fractalkine** | | 0.83 | 0.827 | **63.65**  (28.00-154.78) | **70.07**  (26.17-141.48) | **87.70**  (40.61-550.87) | 0.13 | 0.242 |
| **IP-10** | | 0.17 | 0.269 | **257.55**  (200.00-355.11) | **237.23**  (183.36-311.03) | **309.98**  (224.30-627.10) | 0.01 | 0.091 |
| **GIP** | | 0.20 | 0.269 | **80.75**  (29.5-202.8) | **72.3**  (26.8-158.0) | **119.7**  (27.8-250.9) | 0.19 | 0.263 |
| **GLP** | | 0.58 | 0.620 | **63.3**  (32.4-102.3) | **56.2**  (33.70-91.0) | **43.7**  (30.0-101.4) | 0.69 | 0.739 |
| **MMP-3** | | 0.21 | 0.269 | **5031.2**  (2232.6-9900.4) | **6103.1**  (2092.7-14344.4) | **3898.0**  (2429.2-8987.9) | 0.45 | 0.523 |
| **MMP-9** | | 0.01 | 0.056 | **31475.7**  (13584.3-114664.5) | **19873.0**  (10778.6-40773.3) | **17159.1**  (7351.3-79611.6) | 0.80 | 0.796 |
| **IL-6** | | 0.37 | 0.431 | **8.0**  (0.9-13.7) | **8.0**  (1.0-13.7) | **12.7**  (1.0-13.7) | 0.04 | 0.147 |
| **G-CSF** | | 0.21 | 0.269 | **26.8**  (16.4-44.8) | **24.1**  (16.8-37.3) | **39.0**  (19.7-46.2) | 0.09 | 0.238 |
| **BP**  mmHg | **Systolic** | 0.06 | 0.171 | **119**  (110-128) | **114**  (108-122) | **120**  (109-130) | 0.33 | 0.420 |
|  | **Diastolic** | 0.01 | 0.061 | **74**  (70-80) | **70**  (68-80) | **76**  (69-80) | 0.19 | 0.263 |
| **Insulin** pmol/mL | | 0.18 | 0.269 | **1.2**  (0.5-2.3) | **1.0**  (0.5-1.7) | **1.6**  (1.0-2.9) | 0.01 | 0.091 |
| **BMI,** kg/m^2^ | | 0.02 | 0.063 | **28.4**  (26.4-30.7) | **28.0**  (26.0-30.3) | **28.5**  (26.6-30.9) | 0.14 | 0.242 |
| **Median**(IQR), Analytes in pg/mL**; ***GERD vs Controls by Mann-Whitney U  **^†^**BE vs Controls by Mann-Whitney U  ******Benjamini-Hochberg Critical Value | | | | | | | | |
